# From Rights to Responsibilities at Work: The Longitudinal Interplay of Decent Work, Flourishing, and Job Performance Across Italian Employees

**DOI:** 10.3390/bs15040499

**Published:** 2025-04-09

**Authors:** Ivan Marzocchi, Luigi Fusco, Ilaria Olivo, Stefano Isolani, Francesca Spinella, Valerio Ghezzi, Monica Ghelli, Matteo Ronchetti, Benedetta Persechino, Claudio Barbaranelli

**Affiliations:** 1 Department of Psychology, University of Rome La Sapienza, 00185 Rome, Italy; luigi.fusco@uniroma1.it (L.F.); ilaria.olivo@uniroma1.it (I.O.); stefano.isolani@uniroma1.it (S.I.); francesca.spinella@uniroma1.it (F.S.); valerio.ghezzi@uniroma1.it (V.G.); claudio.barbaranelli@uniroma1.it (C.B.); 2 Department of Occupational and Environmental Medicine, Epidemiology and Hygiene, Italian Workers’ Compensation Authority (INAIL), Monte Porzio Catone, 00078 Rome, Italy; m.ghelli@inail.it (M.G.); m.ronchetti@inail.it (M.R.); b.persechino@inail.it (B.P.)

**Keywords:** decent work, health, gain spirals, resources, prospective study

## Abstract

From a positive psychological standpoint, access to decent work extends beyond fulfilling economic needs: it is a fundamental human right. While significant efforts have been made to examine the societal implications of decent work, surprisingly little attention has been directed toward its impact on individual employees. Integrating the Conservation of Resources theory and the Self-Determination theory, this study aims to advance understanding of this topic by exploring the dynamic and reciprocal interplay among decent work, flourishing (namely, an indicator of strong individual well-being), and job performance. Data were collected from 426 Italian employees (62.7% female) by administering a survey in three waves with a one-month lag. A Cross-Lagged Panel Model approach was employed. The findings highlight that (a) decent work is positively associated with later flourishing; (b) flourishing positively affects later job performance; (c) flourishing fully mediates the relationship between decent work and job performance; and (d) the relationship between flourishing and decent work is reciprocal, with flourishing also enhancing the perception of decent work over time. Our study contributes to advancing the understanding of decent work and its implications, demonstrating the importance of promoting a decent work environment to foster flourishing and performance. This creates a mutually reinforcing cycle of well-being and productivity.

## 1. Introduction

Work is not just a routine activity but a defining aspect of human life. The recent transformations in the world of work, which have resulted in a dramatic reduction in both the quantity and quality of available jobs (Blustein et al., 2018), highlight an urgent call to action: the need to promote adequate, healthy, and resourceful work (Nielsen & Christensen, 2021). In that regard, decent work, a concept introduced by the International Labor Organization (ILO, 1999), encapsulates the baseline attributes of adequate and healthy work, including the promotion of rights at work, employment, social protection, and meaningful social dialogue. From a positive psychological perspective, having access to decent work transcends mere economic necessity: It is a fundamental human right that fosters well-being (Blustein et al., 2023). In turn, individuals who experience well-being are also more motivated, creative, and productive (Diener, 2013). It is thus really surprising that, despite the growing importance of ensuring decent work in today’s changing work landscape, no research has yet explored the dynamic and reciprocal relationships between decent work, well-being, and job performance.

In the current study, we seek to address this gap by examining the longitudinal associations between decent work, flourishing (i.e., an indicator of general well-being that reflects satisfaction, optimism, and confidence), and job performance. Our research objectives are threefold: (a) to assess the longitudinal effect of decent work on flourishing; (b) to explore how flourishing influences job performance and mediates the association between decent work and job performance over time; and (c) to investigate the reciprocal, dynamic interplay between decent work, flourishing, and job performance.

Examining the concept of decent work through the perspective of Occupational Health Psychology and at the individual-level is still a relatively new and underdeveloped area of study (Ferraro et al., 2018b). Previous studies on decent work mainly focus on societal-level outcomes (Blustein et al., 2016). We contribute to this emerging field by examining how decent work impacts individuals, focusing on two key indicators: flourishing, representing a well-being indicator that extends above and beyond the work domain, and job performance. To do so, we draw on the Conservation of Resources (COR) theory (Hobfoll et al., 2018), positioning decent work as a key set of resources within the workplace, and explore its relationship with flourishing and job performance, guided by the foundational principles of Self-Determination theory (SDT) (Deci et al., 2017). We adopt a three-wave longitudinal approach, which addresses a significant limitation of most previous studies on decent work, which have been cross-sectional (Blustein et al., 2023). While cross-sectional studies provide valuable insights, they cannot capture the temporal dynamics between variables. Our rigorous longitudinal design overcomes this limitation by examining the dynamic, reciprocal relationship between decent work, flourishing, and job performance, offering a deeper understanding of how these elements influence each other over time. This approach allows us not only to explore how decent work enhances well-being and job performance but also to understand how it fosters a positive feedback loop in which flourishing further strengthens both the perception and the opportunities for working decently.

### 1.1. Decent Work

The concept of decent work was first proposed by the International Labor Organization (ILO, 1999) as a primary goal, encompassing four strategic, person-centered objectives: the promotion of rights at work, employment, social protection, and social dialogue. Decent work has a long-standing history and is now one of the key Sustainable Development Goals for 2030, identified by the United Nations (2015). The International Labor Organization defines decent work as embodying people’s aspirations for their professional lives (ILO, 2015). It encompasses access to productive employment that provides fair remuneration, workplace safety, and social protection for families. Additionally, it promotes opportunities for personal growth and social inclusion and ensures the freedom to voice concerns, participate in decision-making, and organize, while upholding equality of opportunity and treatment for both women and men (ILO, 2015).

Since the concept of decent work was first introduced, numerous scholars and international organizations have worked to enhance its framework and develop more precise methods for its measurement. For example, a more recent psychology-based conceptualization (Ferraro et al., 2018b) suggests seven core aspects that define decent work. The first component, Fundamental Principles and Values at Work, emphasizes that work and the workplace should embody justice, dignity, freedom, acceptance, fairness, trust, clear norms, participation, solidarity, and mental health. These principles are the foundation of the decent work concept and serve as essential pillars (Ferraro et al., 2018b). The second component, Adequate Working Time and Workload, stresses the need to balance work-related demands, such as effort, pace, deadlines, shifts, and schedules, with personal life. The third component, Fulfilling and Productive Work, emphasizes that work should contribute to both personal and professional growth, fostering fulfillment while generating value for the individual and society. The fourth component, Meaningful Remuneration, highlights the importance of fair compensation, ensuring that individuals’ earnings support their autonomy and dignity, as well as that of their families. The fifth component, Social Protection, refers to the safety nets provided by public or private insurance systems to protect employees and their families in case of unemployment, illness, or retirement. Opportunities, the sixth component, focuses on the potential for career advancement, including learning opportunities, benefits, income growth, and professional development. Finally, the seventh component, Health and Safety, encompasses employees’ perceptions of being safeguarded from physical and psychological risks in the workplace (Ferraro et al., 2018b).

### 1.2. Analyzing Decent Work Through the Lens of COR Theory and SDT

COR theory assumes that individuals are motivated to protect their current resources (conservation) and acquire new resources (acquisition). Resources, which can be objects, states, conditions, and other things that people value (Hobfoll et al., 2018), are anything perceived by the individuals to help attain their goals (Halbesleben et al., 2014). Conceptualizing decent work within this framework, the key aspects of decent work align closely with the concept of resources. For example, the components of Fundamental Principles and Values at Work and Adequate Working Time and Workload embody essential resources that help foster a supportive, respectful, and healthy work environment. Moreover, these components also play a vital role in mitigating the negative impacts of workplace psychosocial risks, thereby promoting employee well-being and productivity. The aspects of Fulfilling and Productive Work and Opportunities represent resources that support opportunities for personal and professional growth. Finally, Meaningful Remuneration, Social Protection, and Health and Safety are resources that provide stability, well-being, and security.

Resources have a motivational potential, leading to well-being and, in turn, positive behaviors (Bakker & Demerouti, 2017). This assumption has been confirmed in both cross-sectional and longitudinal research (Lesener et al., 2019; Nielsen et al., 2017). Extending these findings, it is also possible that decent work, as a set of workplace resources, is similarly associated with well-being. The beneficial effects of decent work are likely attributable to its ability to fulfill several basic needs that, consistent with SDT (Deci et al., 2017), are essential for employee motivation: the need for autonomy, competence, and relatedness (Duffy et al., 2016). Indeed, according to SDT, employees are motivated and experience well-being when these fundamental psychological needs are met (Deci et al., 2017). Autonomy reflects the desire for a sense of choice and freedom in one’s actions; competence involves the need to feel effective in interactions with the environment; finally, relatedness pertains to the need to establish connections with others (Deci et al., 2017).

Past studies have demonstrated that decent work is positively related to several work-related well-being indicators, such as work engagement (Graça et al., 2021; Xu et al., 2022) and work motivation (Ferraro et al., 2018a). However, work is a defining and all-encompassing aspect of human life, with the average person spending over 90,000 h at work throughout their lifetime—roughly one-third of their entire existence (Pryce-Jones, 2010). Its impact extends far beyond the office, influencing overall quality of life (Hakanen & Schaufeli, 2012). Thus, in the present study, we propose that the effects of decent work extend beyond the workplace, influencing life experiences. Specifically, we focus on flourishing, a key indicator of general well-being that represents an individual’s optimal functioning (Keyes & Annas, 2009). Flourishing encompasses various aspects of positive functioning, including a sense of meaning and purpose in life, nurturing positive relationships, active engagement in daily activities, contributing to others’ well-being, demonstrating competence, embracing self-acceptance, maintaining optimism, and experiencing respect (Diener et al., 2010). When individuals flourish, they experience positive emotions, demonstrate strong psychological functioning (e.g., dedication, energy, determination, and a sense of meaning and purpose in their life), and excel socially (e.g., through social acceptance, coherence, contribution, integration, and growth) (Diener et al., 2010). Based on these reasonings, and in line with COR theory and SDT, we propose our first hypothesis:

**Hypothesis** **1.** *Decent work is positively associated with later flourishing.*

The happy worker–productive worker thesis suggests that employees experiencing well-being tend to perform at their best (Wright & Cropanzano, 2000). One possible explanation is that these employees benefit from greater energy, enthusiasm, and a deeper level of engagement in their tasks (Kim et al., 2012). Achieving an optimal state of well-being serves as a foundation for positive behavioral outcomes, and job performance is one of them (Bakker & Demerouti, 2008). Job performance refers to the actions and behaviors of employees that are intended to enhance the organization’s effectiveness and contribute to its overall success (Campbell et al., 1990). Job performance is also a pivotal component of several workplace well-being frameworks, which state that well-being is both an antecedent of job performance and a mechanism through which working conditions lead to optimal performance (Bakker et al., 2023).

In a similar vein, we expect flourishing to be a predictor of job performance and a mechanism through which decent work may lead to better job performance. Previous studies on this topic, though limited, have demonstrated that flourishing is linked to key behavioral outcomes, including reduced turnover intention, enhanced organizational citizenship behavior, and improved task performance (Redelinghuys et al., 2019). From the perspective of COR theory, flourishing is thus a valuable resource because it is both a desirable end in itself (representing positive individual functioning) and a means to achieving other desirable outcomes (e.g., optimal job performance) (Hobfoll et al., 2018). Thus, based on these argumentations, we propose our second and third hypotheses:

**Hypothesis** **2.** *Flourishing is positively associated with later job performance;*

**Hypothesis** **3.** *Flourishing at T2 mediates the longitudinal association between decent work at T1 and job performance at T3.*

### 1.3. “Gain Spirals”: Reciprocal Relationships Between Decent Work, Flourishing, and Job Performance

Beyond exploring the role of decent work in fostering flourishing and job performance, we take a further step by examining the reciprocal relationships among these three variables. According to COR theory, “gain spirals” suggest that individuals with a strong initial resource base are better positioned to accumulate additional resources, with early resource gains leading to further positive developments over time (Hobfoll et al., 2018). Applied to our context, an initial state of flourishing could trigger a self-reinforcing cycle, enabling individuals to continuously acquire new resources, including a heightened perception of decent work and even greater levels of flourishing. Feeling well may help individuals to selectively attract, broaden, and maintain attention to positive cues in the work environment (Jiang et al., 2025; Tang, 2014). Thus, we propose that employees who are flourishing may be more attuned to and derive greater benefits from the positive aspects of their work environment. Their heightened well-being may enable them to recognize and internalize supportive workplace cues (e.g., job security, career development opportunities, and fair treatment) more effectively. Moreover, an employee who feels psychologically fulfilled may be more likely to proactively negotiate better working conditions, advocate for professional development opportunities, or create a supportive work environment. This is likely to generate a gain spiral, where flourishing enhances the subsequent perception of decent work, which, in turn, further promotes future flourishing. Thus, we propose the following hypotheses:

**Hypothesis** **4.** *Flourishing is positively associated with later decent work;*

**Hypothesis** **5.** *Decent work mediates the longitudinal association between flourishing at T1 and flourishing at T3.*

Finally, we also explore the reverse effects of job performance on flourishing. Optimal job performance may be positive for the well-being of the employee since high performance often leads to recognition, positive feedback, and a sense of accomplishment (Laguna & Razmus, 2019). These positive outcomes not only validate an individual’s efforts but also contribute to a stronger sense of self-worth, job satisfaction, and overall well-being (Laguna & Razmus, 2019). Considering the above, we propose the following hypothesis:

**Hypothesis** **6.** *Job performance is positively associated with later flourishing.*

## 2. Materials and Methods

### 2.1. Procedure and Participants

Participants were recruited through a snowball sampling strategy. The research assistants contacted employees from various organizations, inviting them to propose the survey to their colleagues. The eligibility criteria were as follows: (a) being at least 18 years of age and (b) being native Italian speakers. After they ascertained their availability to participate in the research project, they distributed the survey link to employees within their organizations, who had five days to complete it. Participation was voluntary, anonymous, and not rewarded by any incentive. An online-based self-reported questionnaire was administered through the *SurveyMonkey* platform. Data processing and privacy protection were guaranteed following the GDPR Regulation Framework issued by the European Union (2016). The survey was administered in three waves, with a one-month interval between each wave.

The total sample consisted of *n* = 426 Italian employees^1^ between the ages of 18 and 68 (M = 44.07, SD = 13.25), of whom 65.3% (*n* = 267) were females. About their maximum educational level, most of them had completed high school (43.4%; *n* = 184), followed by a master’s degree (29.7%; *n* = 126), and a bachelor’s degree (13.9%; *n* = 59). Most participants worked in the education (23.3%; *n* = 85), healthcare (8.8%; *n* = 35), and hospitality sectors (8.5%; *n* = 31). Regarding their occupational characteristics, most of the sample had a permanent contract (76.3%; *n* = 322) while 13.3% (*n* = 56) had a fixed-term contract and 4.0% (*n* = 17) were self-employed. Furthermore, 79% (*n* = 335) were full-time workers. Finally, 81.6% (*n* = 328) were office-based workers, 17.4% (*n* = 70) were hybrid workers, and only 1.0% (*n* = 4) were remote workers. The complete descriptive statistics are reported in Table 1. Before conducting the main analyses, we performed a preliminary check on missing data. Specifically, we conducted Little’s MCAR test, which assesses whether missing values are completely at random. The results of this test indicated that non-response was completely random (Little’s test: χ^2^ (2232) = 2248.49, *p* = 0.40). Consequently, following methodological recommendations to reduce bias and maintain statistical power in longitudinal studies (Graham, 2009), we implemented full information maximum likelihood (FIML) estimation. This technique uses all available data to produce unbiased and efficient parameter estimates under conditions of ignorable missingness (Enders & Bandalos, 2001).

### 2.2. Measures

Decent work was assessed through the Decent Work Questionnaire (Ferraro et al., 2021). This tool is based on a 5-point Likert scale (1 = I do not agree to 5 = I completely agree) and assesses Decent Work through the seven dimensions described above (Ferraro et al., 2018a). For the present study, an eight-item reduced version of the scale was used (see the Supplemental Material and Table S1 for additional information). An item sample was “I feel that I am protected if I become unemployed (unemployment insurance, government benefits, social programs, etc.)”. McDonald’s ω reliability coefficients were ω = 0.79 at T1, ω = 0.82 at T2, and ω = 0.85 at T3.

Flourishing was investigated through the Flourishing Scale (Diener et al., 2010), an eight-item measure based on a 7-point Likert scale (1 = strongly disagree to 7 = strongly agree). An item sample was “My social relationships are supportive and rewarding”. For the present study, McDonald’s ω reliability coefficients were ω = 0.90 at T1, ω = 0.91 at T2, and ω = 0.94 at T3.

Job performance was assessed through a single-item measure (Kessler et al., 2003). Based on an 11-point Likert scale, this item asked participants to rate the quality of their job performance during the last month (0 = worst job performance to 10 = best job performance).

Covariates: Gender and age were included as covariates due to their potential influence on health at work and performance (Wilks & Neto, 2013). Indeed, a substantial body of literature has linked gender to various health indicators, including work-related stress and burnout. Research suggests that men and women may exhibit different vulnerabilities to specific types of occupational strain (Purvanova & Muros, 2010). At the same time, age has been negatively associated with burnout (Marchand et al., 2018) and positively linked to various dimensions of job performance, likely due to accumulated experience and skill development over time (Ng & Feldman, 2008).

### 2.3. Analytic Strategy

All analyses were conducted using Mplus Version 8.10 (Muthén & Muthén, 2017). Given the hierarchical data structure (individuals nested within organizations), we utilized the “type  =  complex” command to adjust standard errors and significance tests, accounting for the non-independence of observations.

Since the decent work and flourishing scales included more than five items, we adopted a partially disaggregated method by employing an item-parceling strategy. This approach involves averaging multiple items that measure the same construct (Coffman & MacCallum, 2005). The parceling strategy offers several benefits compared to using single items, such as reducing the number of parameters’ estimates and minimizing sources of sampling error (Little et al., 2022).

As a preliminary step, we tested the longitudinal invariance of decent work, flourishing, and job performance using the partial saturation approach (Little, 2013). Specifically, we first implemented an unconstrained model (i.e., configural invariance) in which no constraints across time were imposed on any parameter. Second, we constrained factor loadings to be equal across waves (i.e., metric invariance). Third, we constrained observed intercepts to be equal across waves (i.e., scalar invariance).

We tested the hypothesized relationships among the variables using a cross-lagged panel model with lag-2 effects (CL2PM) (Lüdtke & Robitzsch, 2022). Differing from the classic CLPM, this approach posited a higher-order autoregressive effect from T1 to T3: positing a higher-order autoregressive path captures long-term stability or influence of a variable across non-adjacent waves. As the intervals between waves had the same length (one month), we adopted cross-wave equality constraints on structural coefficients (i.e., autoregressive and cross-lagged effects). This was performed because we did not expect systematic differences in the structural coefficients across intervals (Cole & Maxwell, 2003; Little et al., 2007). A preliminary check of the tenability of these constraints was supported on both autoregressive and cross-lagged effects (see the Supplemental Material, Table S3).

**Figure 1:**
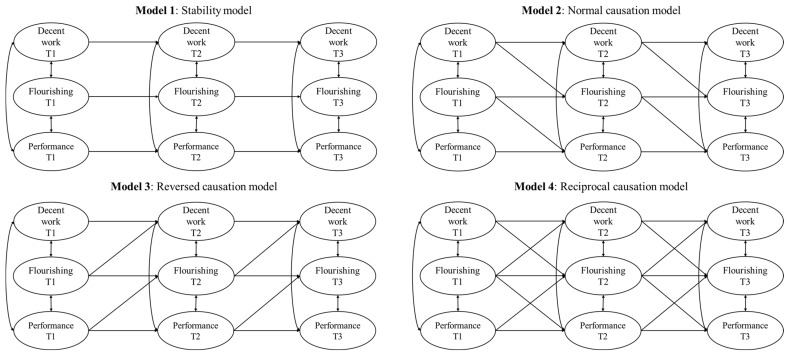
Tested models.

To investigate our hypotheses, we compared a sequence of nested models (Figure 1). We first estimated a model in which only the autoregressive pathways between the variables were specified (Model 1). This model was compared to one in which all hypothesized normal causation pathways were added (Model 2). We also evaluated the fit of a reversed (Model 3) and reciprocal causation model (Model 4). Synchronous correlations between the three constructs in the same wave were allowed in all models. We compared the four different models considering the following indices and cut-off scores (Hu & Bentler, 1999): Comparative Fit Index (CFI ≥ 0.90), Tucker–Lewis Index (TLI ≥ 0.90), Root Mean Square Error of Approximation (RMSEA ≤ 0.08), and Standardized Root Mean Square Residual (SRMR ≤ 0.08). Specifically, we relied on the guidelines proposed by Cheung and Rensvold (2002) and Chen (2007), according to which changes in CFI < 0.01, RMSEA < 0.015, and SRMR < 0.030 indicate an improvement in model fit. Direct and indirect effects were estimated and reported based on the best-fitting model. This was finally enriched with potentially relevant covariates (i.e., gender and age), an approach that helps isolate the true effects of the variables under study and reduces the risk of spurious findings due to common method bias (Podsakoff et al., 2012).

## 3. Results

Table 2 reports mean values, standard deviations, skewness, kurtosis, and correlations for the variables at the three different time points. All variables, except job performance, had non-problematic levels of skewness and kurtosis. Consequently, we employed the robust maximum likelihood estimator (MLR) in the main analysis, as it is robust to non-normal data. All of the correlations between the variables investigated were significant and in the expected directions.

Preliminarily, the test of longitudinal invariance on decent work, flourishing, and job performance showed no differences between the fit of the configural, metric, and scalar measurement invariance models. Thus, scalar longitudinal invariance was achieved in the three variables. The results of these analyses are reported in the Supplemental Material (Table S2).

CFA demonstrated that the measurement model fitted the data well: Yuan–Bentler (YB)χ^2^ (241) = 443.74, CFI = 0.961, TLI = 0.955, RMSEA = 0.044 (95% CIs: 0.038–0.051, *p* = 0.921), and SRMR = 0.058. Thus, we compared a series of nested models to identify the most appropriate pathways explaining the relationships among our three variables. As shown in Table 3, Model 4 (i.e., the reciprocal causation model) exhibited the best goodness-of-fit compared to the stability, causal, and reverse models, as all fit indices improved and did not drop below the suggested cutoff values of CFI < 0.01, RMSEA < 0.015, and SRMR < 0.030. Consequently, this was retained as our final model. The model fit was also satisfactory after the inclusion of covariates (i.e., age and gender): YBχ^2^ (345) = 623.171, CFI = 0.951, TLI = 0.943, RMSEA = 0.044 (95% CIs: 0.038–0.049, *p* = 0.976), and SRMR = 0.068.

**Figure 2:**
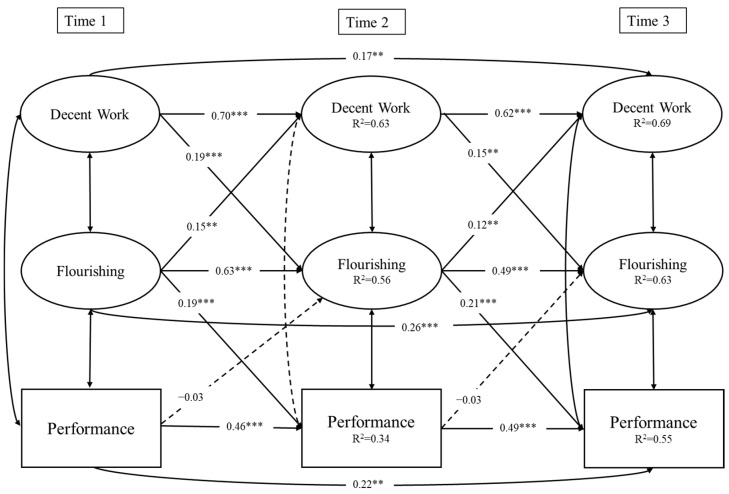
Results of the reciprocal causation model. Note: Dotted lines denote non-significant paths. The results are controlled for age and gender. Intra-wave correlations among the variables were specified in the model: At T1, decent work correlated with flourishing (0.57 ***) and job performance (0.27 ***), and flourishing correlated with job performance (0.41 ***). At T2, decent work correlated with flourishing (0.41 ***) but not with job performance (0.17), and flourishing correlated with job performance (0.24 **). Finally, at T3, decent work correlated with flourishing (0.36 ***) and job performance (0.25 **), and flourishing correlated with job performance (0.22 *). *** *p* < 0.001; ** *p* < 0.01; * *p* < 0.05.

The results of the reciprocal causation model are shown in Figure 2. In line with Hypothesis 1, decent work at T1 and T2 positively influenced flourishing at T2 (β = 0.19, *p* < 0.001) and T3 (β = 0.15, *p* < 0.01), respectively. In line with Hypothesis 2, flourishing at T1 and T2 positively influenced job performance at T2 (β = 0.19, *p* < 0.001) and T3 (β = 0.21, *p* < 0.001), respectively. Consistent with Hypothesis 3, decent work at T1 was significantly associated with job performance at T3 through flourishing at T2 (effect size = 0.036, 95% CI [0.008, 0.063]). Hypotheses 4 and 5 were also supported, as flourishing at T1 and T2 was associated with decent work at T2 (β = 0.15, *p* < 0.01) and T3 (β = 0.12, *p* < 0.01), respectively; moreover, flourishing at T1 was significantly associated with flourishing at T3 through decent work at T2 (effect size = 0.022, 95% CI [0.003, 0.041]). Finally, job performance at T1 and T2 was not associated with flourishing at T2 (β = −0.03, *p* = 0.47) and T3 (β = −0.03, *p* = 0.47), respectively; thus, Hypothesis 6 was not supported.

All variables showed significant autoregressive paths across time points, suggesting that decent work, flourishing, and job performance were fairly stable across waves. Among the covariates, the effects were generally non-significant, with the exception of the path from gender to flourishing at T1 (β = −0.13, *p* < 0.05). Specifically, female employees reported lower levels of flourishing at T1 compared to their male counterparts. Overall, the model explained 63.4% of the variance of decent work, 56.1% of flourishing, and 34.1% of job performance at T2, and 71.2% of the variance of decent work, 63.4% of flourishing, and 54.7% of job performance at T3.

## 4. Discussion

Work is a fundamental part of life, shaping our daily routines, personal growth, and sense of purpose. In recent years, the concept of decent work has gained prominence in research (Blustein et al., 2023). Decent work is a concept that outlines the fundamental characteristics of work, aligning with the International Labor Organization’s (ILO) four strategic objectives: promoting rights at work, ensuring employment opportunities, providing social protection, and fostering social dialogue (ILO, 1999). Despite its relevance, surprisingly little effort has been devoted to understanding its individual-level outcomes, especially in a longitudinal way (Blustein et al., 2023). In the current study, we seek to address some of the gaps in the existing literature by investigating the longitudinal, reciprocal relationships between decent work, flourishing, and job performance. In doing so, we drew upon the core principles of COR theory (Hobfoll et al., 2018), framing decent work and its features as a set of workplace resources available to employees.

Due to its all-encompassing nature, work extends its influence beyond the workplace, shaping various aspects of an individual’s life and well-being (Hakanen & Schaufeli, 2012). For this reason, our study explored whether engaging in decent work, with all that it entails, enables individuals to “flourish” in their overall lives, rather than restricting our investigation solely to work-related outcomes. When individuals flourish, they experience overall well-being, exhibit strong psychological functioning (e.g., dedication, vitality, perseverance, and a deep sense of purpose), and thrive socially (Diener et al., 2010). Our results highlight that decent work is an antecedent of flourishing (Hypothesis 1). Thus, by offering essential resources such as strong work values, opportunities for professional growth, fair compensation, social protection, and a balanced work pace, decent work shapes individuals’ positive functioning. Indeed, consistent with SDT (Deci et al., 2017), decent work allows individuals to satisfy basic psychological needs, such as autonomy, competence, and relatedness. This cultivates a sense of meaning and purpose in life, strengthens supportive relationships, enhances engagement in daily activities, promotes self-acceptance, and fosters optimism (Diener et al., 2010).

In turn, flourishing promotes optimal job performance (Hypothesis 2) and mediates the association between decent work and job performance (Hypothesis 3). Thus, offering adequate and fair working conditions is not only crucial for well-being but also has significant implications for organizational productivity. These results corroborate the happy worker–productive worker thesis (Wright & Cropanzano, 2000), demonstrating that employees who flourish in their lives may perform better. Despite the fact that this assumption may seem obvious, previous research has often characterized a “happy worker” as someone who is satisfied or engaged with their job while largely overlooking the broader concept of well-being as reflected in flourishing (Erdogan et al., 2012). Moreover, although high levels of well-being have been shown to be related to several positive outcomes, such as productivity, the relationship of flourishing with these outcomes has been mainly unexplored (Demerouti et al., 2015). Our findings indicate that individuals who feel better about their lives tend to be more effective, likely due to their higher motivation, open-mindedness, creativity, and resilience in handling challenges (Erdogan et al., 2012).

Consistent with Hypotheses 4 and 5, there is a reciprocal association between decent work and flourishing. This finding provides support for the principle of gain spirals from COR theory (Hobfoll et al., 2018). COR theory states that people are motivated to continually gain resources and that they do so by strategically investing resources; this leads to even greater gains (Hobfoll et al., 2018). Previous studies have found gain spirals between resources and positive outcomes, such as work engagement (Mäkikangas et al., 2010; Xanthopoulou et al., 2009). Similarly, our study demonstrates that flourishing can initiate a self-reinforcing cycle where individuals continue to gain resources. When individuals feel well, they are more likely to focus on and amplify the positive aspects of their work environment (Jiang et al., 2025; Tang, 2014). Thus, we suggest that flourishing employees may be better able to benefit from the positive elements in their workplace, such as job security, opportunities for career advancement, and fair treatment. Furthermore, individuals who feel psychologically fulfilled are more likely to take proactive steps to negotiate for improved working conditions, advocate for professional growth, and foster a positive work environment (Hakanen et al., 2018). This sense of well-being empowers them to engage actively with their workplace, leading to a virtuous cycle where both flourishing and decent work are continuously reinforced (as confirmed by Hypothesis 5).

It is worth noting that Hypothesis 6, which stated a reversed effect from job performance to flourishing, was not supported. Job performance is mainly examined as a result of employee well-being, influenced by favorable working conditions (Bakker et al., 2023). However, while research on this aspect is limited, some evidence suggests that job performance can also enhance employee well-being, indicating a bidirectional relationship rather than a solely one-way effect. For example, optimal job performance may drive job satisfaction responses, especially through rewards, positive feedback, and a sense of accomplishment (Laguna & Razmus, 2019; Vecchio et al., 2010). However, in our study, job performance was not associated with later flourishing. Assuming that future research is further needed to elucidate this association, one possible explanation could be that job performance is more closely related to immediate, work-specific outcomes such as work engagement (Laguna & Razmus, 2019). Instead, flourishing may require a deeper level of psychological fulfillment.

### 4.1. Practical Implications

The findings of our study highlight the importance of creating decent work conditions as a way to enhance both well-being and job performance. A higher level of flourishing strengthens employees’ contribution to the organizational purpose: when employees experience well-being in a holistic sense, they are more likely to perform better and contribute positively to the workplace (Wright & Cropanzano, 2000). Thus, this research confirms the importance of designing human resource management policies and practices aligned with the Decent Work concept. Organizations and policymakers should prioritize fair wages, job security, career development opportunities, and a healthy work–life balance, as these factors contribute to overall flourishing. A risk management approach (Leka & Kortum, 2008) that systematically identifies potential harmful working conditions (e.g., psychosocial risks) could serve as a crucial first step in designing targeted interventions aimed at enhancing motivational factors (e.g., job resources and elements of decent work) and ultimately improving employee well-being. Furthermore, organizations should recognize that decent work and flourishing exist in a mutually reinforcing cycle (Hobfoll et al., 2018). This means that investing in employees’ well-being is not just an ethical responsibility but also a strategic move to create a more engaged, proactive, and productive workforce. Employers can support this process by fostering a positive organizational culture, encouraging open communication, and providing opportunities for employees to take an active role in shaping their work environment.

In broader terms, regulatory developments will help better define the scope of action in various areas. In Italy, workplace safety regulations have evolved to strengthen employee health, safety, and protection, particularly in high-risk environments. For example, Law 113/2020, introduced in the healthcare sector, has paved the way for increasing safeguards against workplace aggression, setting an important precedent for future legislative advancements in employee protection. When designing corporate welfare initiatives, it is also crucial to consider the specific characteristics of different labor markets. The Italian labor market, for example, is characterized by a high prevalence of small- and medium-sized enterprises (SMEs). These organizations often have limited structural complexity, which may affect the feasibility and effectiveness of certain well-being initiatives. Therefore, in addition to tailoring interventions to different sectors (especially those with higher risks of poor well-being), there is a need to adapt strategies based on company size. Conducting a thorough assessment of critical issues within SMEs is essential to ensure that well-being policies are not only applicable but also impactful in different organizational contexts.

### 4.2. Study Limitations and Future Research

Our study is not exempt from limitations that should be acknowledged. First, decent work was assessed using a shortened eight-item version of the Decent Work Questionnaire (Ferraro et al., 2018b). While this measure demonstrated reliability, it did not allow for a nuanced examination of how different dimensions of decent work uniquely influence later flourishing and performance. Future studies should consider using the full version of the scale or alternative validated tools (Duffy et al., 2017) to provide a more comprehensive assessment of decent work and its effects.

Second, we collected all information using self-reported measures; thus, it is possible that common method bias and social desirability may have affected our results, particularly in the assessment of job performance (Podsakoff et al., 2003). To mitigate these concerns, future research could incorporate objective performance indicators (e.g., sales performance, absenteeism, and return on investment) and multi-source data collection (e.g., supervisor and peer evaluations and customer satisfaction) to enhance the robustness of findings.

Third, the considerable heterogeneity of the sample prevented us from conducting more detailed analyses of differences across work sectors and job types. We recommend addressing this issue in future studies to determine the extent to which our empirical findings apply across various sectors.

Fourth and finally, employees were enrolled within a single national context (i.e., Italy), which limits the generalizability of our findings, and with a snowball sampling strategy. To mitigate some of the concerns linked to our recruiting strategy, we implemented several measures to ensure data quality, such as incorporating multiple attention checks in the surveys and recording data collection timestamps. Notably, while concerns about this recruiting approach have been raised (Marcus et al., 2017), recent meta-analytic evidence suggests that results obtained through snowball sampling do not significantly differ from those based on other sampling methods (Wheeler et al., 2014; Winton & Sabol, 2022) and may even lead to more heterogeneous samples (Demerouti & Rispens, 2014). Nevertheless, future research would benefit from larger, more diverse, and cross-national samples recruited using alternative data collection methods to enhance the robustness and external validity of our results. Additionally, future research would benefit from systematically comparing different time lags to refine our understanding of how temporal dynamics shape decent work and occupational health outcomes as well as well-being and performance.

## 5. Conclusions

This study provides further insights into the reciprocal relationships between decent work, flourishing, and job performance, contributing to a deeper understanding of how adequate working conditions shape both individual well-being and organizational outcomes. Our findings show that decent work serves as a critical set of resources that fosters employees’ overall flourishing, which, in turn, enhances job performance. Moreover, our study reinforces the idea of a virtuous cycle between decent work and flourishing, in line with the gain spiral principle. This underscores the importance of improving work conditions that not only prevent harm but also actively promote personal growth, fulfillment, and proactivity.

## Figures and Tables

**Table 1:** Demographic characteristics.

	Mean (*n*)	SD (%)
**Age**	44.07	13.25
**Sex**		
Male	(137)	(33.5)
Female	(267)	(65.3)
**Educational level**		
Elementary school	(1)	(0.2)
High school	(19)	(4.5)
High School	(184)	(43.4)
Bachelor’s degree	(59)	(13.9)
Master’s degree	(126)	(29.7)
Second-level master’s degree	(25)	(5.9)
Doctorate degree	(10)	(2.4)
**Work sector**		
Manufacturing	(10)	(2.7)
Construction	(4)	(1.1)
Wholesale trade	(22)	(6)
Hospitality	(31)	(8.5)
Communication	(9)	(2.5)
Financial services	(11)	(3)
Business service providers	(15)	(4.1)
ICT	(3)	(0.8)
Education	(85)	(23.3)
Healthcare and social services	(35)	(8.8)
Science and technology	(10)	(2.7)
Artistic and literary fields	(6)	(1.6)
Military service	(25)	(6.8)
Other	(102)	(27.9)
**Seniority**	19.27	11.52
**Role tenure**	11.57	9.68
**Years of work in the current team**	7.44	7.38
**Work contract**		
Permanent	(322)	(76.3)
Fixed-term	(56)	(13.3)
Self-employed	(17)	(4.0)
Other	(27)	(6.4)
**Work hours**		
Full-time	(335)	(79.0)
Part-time	(89)	(21.0)
**Work modality**		
Office-based	(328)	(81.6)
Remote	(4)	(1.0)
Hybrid	(70)	(17.4)

Note: *n* and percentage are shown in parentheses.

**Table 2:** Descriptive statistics, zero-order correlations, and reliability coefficients for the study variables.

	Mean	SD	Skew	Kurt	1.	2.	3.	4.	5.	6.	7.	8.	9.	10.	11.
1. Decent work T1	3.57	0.72	−0.29	0.08	*0.79*										
2. Decent work T2	3.56	0.71	−0.36	−0.22	0.74 ***	*0.82*									
3. Decent work T3	3.61	0.74	−0.23	−0.25	0.66 ***	0.79 ***	*0.85*								
4. Flourishing T1	4.98	0.98	−0.51	0.95	0.46 ***	0.46 ***	0.44 ***	*0.90*							
5. Flourishing T2	4.94	0.99	−0.28	0.99	0.50 ***	0.60 ***	0.54 ***	0.72 ***	*0.91*						
6. Flourishing T3	4.92	1.08	−0.34	0.51	0.36 ***	0.50 ***	0.56 ***	0.67 ***	0.70 ***	*0.94*					
7. Performance T1	8.83	1.28	−0.74	2.59	0.22 ***	0.21 **	0.28 ***	0.38 ***	28 ***	0.25 ***	-				
8. Performance T2	8.82	1.34	−1.45	5.69	0.29 ***	0.35 ***	0.33 ***	0.44 ***	0.45 ***	0.33 ***	0.54 ***	-			
9. Performance T3	8.83	1.36	−1.69	8.33	0.16 *	0.24 **	0.35 ***	0.32 ***	0.38 ***	0.37 ***	0.55 ***	0.60 ***	-		
10. Gender _(0=m;1=f)_	-	-	-	-	−0.11 *	−0.12 *	−0.07	−0.13 **	−0.16 **	−0.10	−0.02	−0.09	−0.02	−	
11. Age	44.07	13.25	−0.23	−1.14	0.14 **	0.17 **	0.13 *	0.03	0.09	0.02	0.04	0.13 *	0.02	−0.11 *	−

Note: SD = standard deviation; Skew = skewness; Kurt = kurtosis. McDonald’s omega coefficients are reported along the principal diagonal (*italicized*). *** *p* < 0.001, ** *p* < 0.01, and * *p* < 0.05.

**Table 3:** Models’ comparison.

	YBχ^2^	df	CFI	TLI	RMSEA (95% CIs)	SRMR	Comparison	ΔCFI	ΔRMSEA	ΔSRMR
M1. Stability	589.89 ***	310	0.949	0.943	0.046 (0.040–0.052, *p* = 0.874)	0.093	-	-	-	-
M2. Normal causation	547.52 ***	306	0.956	0.950	0.043 (0.037–0.049, *p* = 0.977)	0.063	M2 vs. M1	−0.007	−0.003	−0.030
M3. Reversed causation	577.66 ***	306	0.955	0.944	0.044 (0.040–0.051, *p* = 0.894)	0.063	M3 vs. M2	−0.006	−0.002	−0.030
M4. Reciprocal causation	536.03 ***	302	0.958	0.951	0.043 (0.037–0.048, *p* = 0.981)	0.059	M4 vs. M1	−0.009	−0.003	−0.034
							M4 vs. M2	−0.002	0.000	−0.004
							M4 vs. M3	−0.003	−0.001	−0.004

Note: YB = Yuan–Bentler; df = degrees of freedom; CI = confidence intervals; M = model; *** *p* < 0.001.

## Data Availability

The data supporting the findings of this study are available from the corresponding author upon request.

## Note

1 Following the observations of an anonymous reviewer, we conducted a post hoc RMSEA-based power analysis to assess whether our sample size was adequate for the intended analyses using the web-based interface power4SEM (Jak et al., 2021). With 241 degrees of freedom, a sample of 426 participants provided sufficient power to reject an RMSEA of 1 under the hypotheses of close fit (H0 = 0.05; H1 = 0.08), not-close fit (H1 = 0.01), and exact fit (H1 = 0). These results indicate that our sample size is sufficient to ensure robust statistical power in our analyses.

## Supplementary Materials

The following supporting information can be downloaded at: https://www.mdpi.com/article/10.3390/bs15040499/s1.

## Abbreviations

The following abbreviations are used in this manuscript:

COR	Conservation of Resources
SDT	Self-determination theory
