# Supplementary material for: From Rights to Responsibilities at Work: The Longitudinal Interplay of Decent Work, Flourishing, and Job Performance Across Italian Employees
Source: Behav Sci (Basel). 2025 Apr 9;15(4):499. doi: 10.3390/bs15040499 (PMC12023954; doi:10.3390/bs15040499)
Supplement: Supplementary file 1 [file behavsci-15-00499-s001.zip › behavsci-3515792-supplementary.pdf]

SUPPLEMENTAL MATERIAL

Decent work scale

To measure decent work, for the present study we used a reduced version of the original scale (Ferraro et al., 2021). This short version is made up of eight items, covering all the key facets of decent work. As shown in Table S1, the "Social protection" component includes two items instead of one to capture both unemployment and health protection. The items were selected based on their factor loadings (Ferraro et al., 2021).

Table S1: Decent work items and factor loadings from the scalar invariance model

Item	Standardized Factor loadings			Decent work facet
	T1	T2	T3	
Through my work I can develop myself professionally<br>*Attraverso il mio lavoro cresco professionalmente*	.52	.58	.59	Fulfilling
I have all the resources and support that I need to work safely<br>*Ho tutto ciò di cui ho bisogno per lavorare in sicurezza*	.64	.67	.75	Health and safety
I think that I have prospects for improving my salary/benefits<br>*Penso di avere delle possibilità di migliorare il mio salario/la mia retribuzione/ benefici lavorativi*	.50	.52	.59	Opportunities
At my work/job, I am treated with dignity<br>*Nel mio lavoro sono trattata/o con dignità*	.65	.70	.72	Principles
What I earn through my work allows me to live my life with dignity and independence<br>*Quello che guadagno con il mio lavoro mi permette di vivere con dignità e autonomia*	.64	.71	.68	Remuneration
I feel that I am protected if I become unemployed (unemployment insurance, government/social benefits, social programmes, etc.)<br>*Penso di essere tutelata/o se rimanessi senza lavoro (cassa integrazione, sussidi sociali, programmi sociali, ecc.)*	.51	.56	.60	Social protection
I feel that I am protected if I become ill (social security or equivalent, NHS, public aid, health insurance, etc.)<br>*Penso che sarei tutelata/o nel caso mi dovessi ammalare (assistenza sociale, assistenza sanitaria, ecc.)*	.53	.54	.59	Social protection
I consider the pace of my work/job to be appropriate.<br>*Considero adeguato il ritmo di lavoro che la mia occupazione esige*	.54	.57	.60	Time/Load
**Omega coefficients**	.79	.82	.85	

Longitudinal invariance

A precondition to test longitudinal effects is measurement invariance (Little, 2013). Thus, preliminarily, we tested the longitudinal invariance of decent work, flourishing and job performance using the partial saturation approach (Little, 2013). In specific, we implemented an unconstrained model (i.e. configural invariance) in which no constraints across time were imposed on any parameter. Second, we constrained factor loadings to be equal across waves (i.e. metric invariance). Third, we constrained observed intercepts (i.e. scalar invariance). We compared the three different models considering the following indices and cut-off scores (Hu & Bentler, 1999): Comparative Fit Index (CFI ≥ 0.90), Tucker–Lewis Index (TLI ≥ 0.90), Root Mean Square Error of Approximation (RMSEA ≤ 0.08), and Standardized Root Mean Square Residual (SRMR ≤ 0.08). In specific, we relied on the guidelines proposed by Cheung & Rensvold (2002) and Chen (2007), according to which changes smaller than CFI < .01, RMSEA < .015, and SRMR < .030 indicate that the more restrictive model and the less restricted model were equivalent. This approach was favored over the χ^2 difference test, as the χ^2 statistic is highly sensitive to sample size, making even minor model misfit appear statistically significant in moderate to large samples, as was the case in our study (Chen, 2007). The test of longitudinal invariance on

decent work, flourishing, and job performance showed no differences between the fit of the configural, metric, and the scalar measurement invariance models (Table S1). Thus, scalar longitudinal invariance was achieved in the three variables.

Table S2. Results of longitudinal invariance

	YBχ^2	df	CFI	TLI	RMSEA (95%Cis)	SRMR	Comparison	ΔCFI	ΔRMSEA	ΔSRMR
I1. Configural	395.45***	213	.965	.955	.045 (.038 - .052, p = .890)	.052		-	-	-
I2. Metric	423.22***	225	.962	.953	.045 (.039 - .052, p = .866)	.057	I2 vs I1	.003	.000	.005
I3. Scalar	443.74***	241	.961	.955	.044 (.038 - .051, p = .921)	.058	I3 vs I2	.001	.001	.001

Notes. I = invariance; YB = Yuan – Bentler; df = degrees of freedom; Cis = confidence intervals; ***p < .001.

Constraining autoregressive and cross-lagged effects to be equal across waves

As the intervals between waves had the same length (one month), we believed there was no reason to expect systematic differences in the structural coefficients across intervals (Cole & Maxwell, 2003; Little et al., 2007). For this reason, we adopted cross-wave equality constraints on structural coefficients (e.g., autoregressive and cross-lagged effects). To preliminary check the tenable of these constraints, we compared the unconstrained model (i.e., in which no constraints across time and effects were imposed on any parameter) with two alternatives model: the first in which only the autoregressive effects of each variable were constrained to be equal; the second in which both the autoregressive and cross-lagged effects were constrained to be equal. Also in this case, the guidelines of Cheung & Rensvold (2002) and Chen (2007) were adopted to compare the models. The results supported the tenable of these constraints on both autoregressive and cross-lagged effects (Table S2).

Table S3. Models' comparison

	YBχ^2	df	CFI	TLI	RMSEA (95% Cis)	SRMR	Comparison	ΔCFI	ΔRMSEA	ΔSRMR
M4. Reciprocal causation model	536.03***	302	.958	.951	.043 (.037 - .048, p =.981)	.059	-	-	-	-
M4.1 M4 + autoregressive paths constrained	534.55***	305	.959	.946	.042 (.037 - .048, p = .992)	.060	M4.1 vs M4	-.001	-.001	.001
M4.2: M4.1 + cross lagged paths constrained	547.33***	309	.957	.943	.043 (.038 - .049, p = .976)	.072	M4.2 vs M4.1	.002	.001	.012

Notes. M = model; YB = Yuan – Bentler; df = degrees of freedom; CIs = confidence intervals; ***p < .001.
